# Identification of candidate PAX2-regulated genes implicated in human kidney development

**DOI:** 10.1038/s41598-021-88743-1

**Published:** 2021-04-27

**Authors:** Yuta Yamamura, Kengo Furuichi, Yasuhiro Murakawa, Shigeki Hirabayashi, Masahito Yoshihara, Keisuke Sako, Shinji Kitajima, Tadashi Toyama, Yasunori Iwata, Norihiko Sakai, Kazuyoshi Hosomichi, Philip M. Murphy, Atsushi Tajima, Keisuke Okita, Kenji Osafune, Shuichi Kaneko, Takashi Wada

**Affiliations:** 1 Department of Nephrology and Laboratory Medicine, Institute of Medical, Pharmaceutical and Health Sciences, Kanazawa University, 13-1 Takara-machi, Kanazawa, Ishikawa 920-8640 Japan; 2 Department of Nephrology, School of Medicine, Kanazawa Medical University, 1-1 Daigaku, Uchinada, Kahoku, Ishikawa 920-0293 Japan; 3 RIKEN Preventive Medicine and Diagnosis Innovation Program, Yokohama, Kanagawa Japan; 4 Division of Genomic Technologies, RIKEN Center for Life Science Technologies, Yokohama, Kanagawa Japan; 5 Department of Bioinformatics and Genomics, Graduate School of Advanced Preventive Medical Sciences, Kanazawa University, Kanazawa, Ishikawa Japan; 6 Molecular Signaling Section, Laboratory of Molecular Immunology, National Institute of Allergy and Infectious Diseases, National Institutes of Health, Bethesda, MD 20892 USA; 7 Center for iPS Cell Research and Application (CiRA), Kyoto University, Kyoto, Japan; 8 Department of System Biology, Institute of Medical, Pharmaceutical and Health Sciences, Kanazawa University, Kanazawa, Ishikawa Japan

**Keywords:** Developmental biology, Genetics, Stem cells, Nephrology

## Abstract

*PAX2* is a transcription factor essential for kidney development and the main causative gene for renal coloboma syndrome (RCS). The mechanisms of PAX2 action during kidney development have been evaluated in mice but not in humans. This is a critical gap in knowledge since important differences have been reported in kidney development in the two species. In the present study, we hypothesized that key human PAX2-dependent kidney development genes are differentially expressed in nephron progenitor cells from induced pluripotent stem cells (iPSCs) in patients with RCS relative to healthy individuals. Cap analysis of gene expression revealed 189 candidate promoters and 71 candidate enhancers that were differentially activated by PAX2 in this system in three patients with RCS with *PAX2* mutations. By comparing this list with the list of candidate Pax2-regulated mouse kidney development genes obtained from the Functional Annotation of the Mouse/Mammalian (FANTOM) database, we prioritized 17 genes. Furthermore, we ranked three genes—*PBX1*, *POSTN*, and *ITGA9*—as the top candidates based on closely aligned expression kinetics with PAX2 in the iPSC culture system and susceptibility to suppression by a Pax2 inhibitor in cultured mouse embryonic kidney explants. Identification of these genes may provide important information to clarify the pathogenesis of RCS, human kidney development, and kidney regeneration.

## Introduction

PAX2 is an essential transcription factor for kidney development. PAX2 is expressed in multiple urogenital tissues, including the nephric duct, cap mesenchyme, and differentiating nephron and collecting duct of the developing kidney^1,2^. In mice, Pax2 is required for the differentiation of the mesenchyme to the epithelium. Therefore, *Pax2*^−/−^ mice completely lack metanephric kidneys in the embryonic period and have no kidneys at birth and die immediately after birth. *Pax2*^+/−^ mice develop kidney hypoplasia and vesicoureteral reflux^3^. In humans, *PAX2* is one of the key disease genes that are defective in renal coloboma syndrome (RCS)^4,5^, which is characterized by kidney hypoplasia or dysplasia and optic nerve dysplasia.

In mice, it is well known that PAX2-regulated gene expression was influenced by many transcriptional and intracellular signaling factors^6^ and epigenetic mechanisms regulated by Pax transactivation domain interacting protein and mixed-lineage leukemia complex^7,8^. Nevertheless, the mechanisms of PAX2 regulation of human kidney development are poorly understood, in part because the human embryonic kidney tissue is not accessible. Recently, methods of inducing kidney lineage cells from induced pluripotent stem cells (iPSCs) have been developed^9,10^. iPSC organoids derived from these cells may become useful in studies of human kidney development.

To identify candidate genes regulated by PAX2 during human kidney development, we performed comprehensive transcriptome analysis of nephrons developing in vitro from iPSCs from patients with RCS and healthy donors by cap analysis of gene expression (CAGE). CAGE is based on the preparation and sequencing of concatamers of DNA tags derived from the initial 20 nucleotides from the 5′ end of mRNAs. CAGE allows high throughput gene expression analysis and accurate profiling of transcription start sites, including promoter and enhancer usage analysis^11^.

In this study, we investigated PAX2-regulated genes in human kidney development using induced kidney lineage cells from RCS patient-derived iPSCs (RCS-iPSCs) and CAGE. Moreover, 189 promoters and 71 enhancers, especially three influential candidate genes, which are conserved in mice and humans, were detected. This knowledge provides new clues for a deeper understanding of PAX2-associated biology in humans, including the molecular pathogenesis of RCS, and mechanisms of human kidney development and regeneration^12,13^.

## Results

### Establishment and characterization of iPSCs derived from patients with RCS

Three unrelated adult Japanese female patients with RCS who were diagnosed with morphological kidney defects, ophthalmologic abnormalities, and family history of *PAX2* gene mutations were selected for the study^14^. Their clinical characteristics are shown in Table 1. Patient 1 has a heterozygous frameshift mutation in exon 2, which is known as the key region for DNA binding. Patients 2 and 3 have heterozygous missense mutations in exon 2. These two single-nucleotide mutations (ex2 c.212G > C, R71T and ex2 c.187G > A, G63S) were suggested to influence the binding to target genes in a previous study^14^. RCS-iPSCs were established by transfection of patient peripheral blood mononuclear cells with episomal vectors encoding *OCT4*, *SOX2*, *KLF4*, *L-MYC*, and *LIN28* and *p53* shRNA. Control iPSCs were previously established from healthy blood donors using the same method.

**Table 1:** Clinical and genetic information of patients with renal coloboma syndrome (RCS).

Case	Age (diagnostic age)	Sex	*PAX2* mutation	Kidney		Ocular fundus
				Morphology	Kidney function	
1	48 (44)	Female	Ex2 c.119-120delGC R40H fsX13	Hypoplasia Atrophy	HD	4
2	43 (34)	Female	Ex2 c.212G > C R71T	Malrotation	HD	6
3	42 (37)	Female	Ex2 c.187G > A G63S	Hypoplasia Atrophy	Cr 2.20	4

The ocular fundus score was evaluated based on the methods described in previous studies^14^.

The score of ocular fundus: Score 0, normal optic disc; Score 1, optic disc dysplasia with an unusual pattern of the retinal vessels and cilioretinal arteries; Score 2, optic disc pit associated with vascular abnormalities and cilioretinal artery; Score 3, large coloboma involving the entire surface of the optic disc; Score 4, large coloboma of the optic disc and adjacent retina or morning glory anomaly (with radial emergence of the retinal vessels)^14^.

*HD* hemodialysis, *Cr* serum creatinine, *Ex* exon.

**Figure 1:**
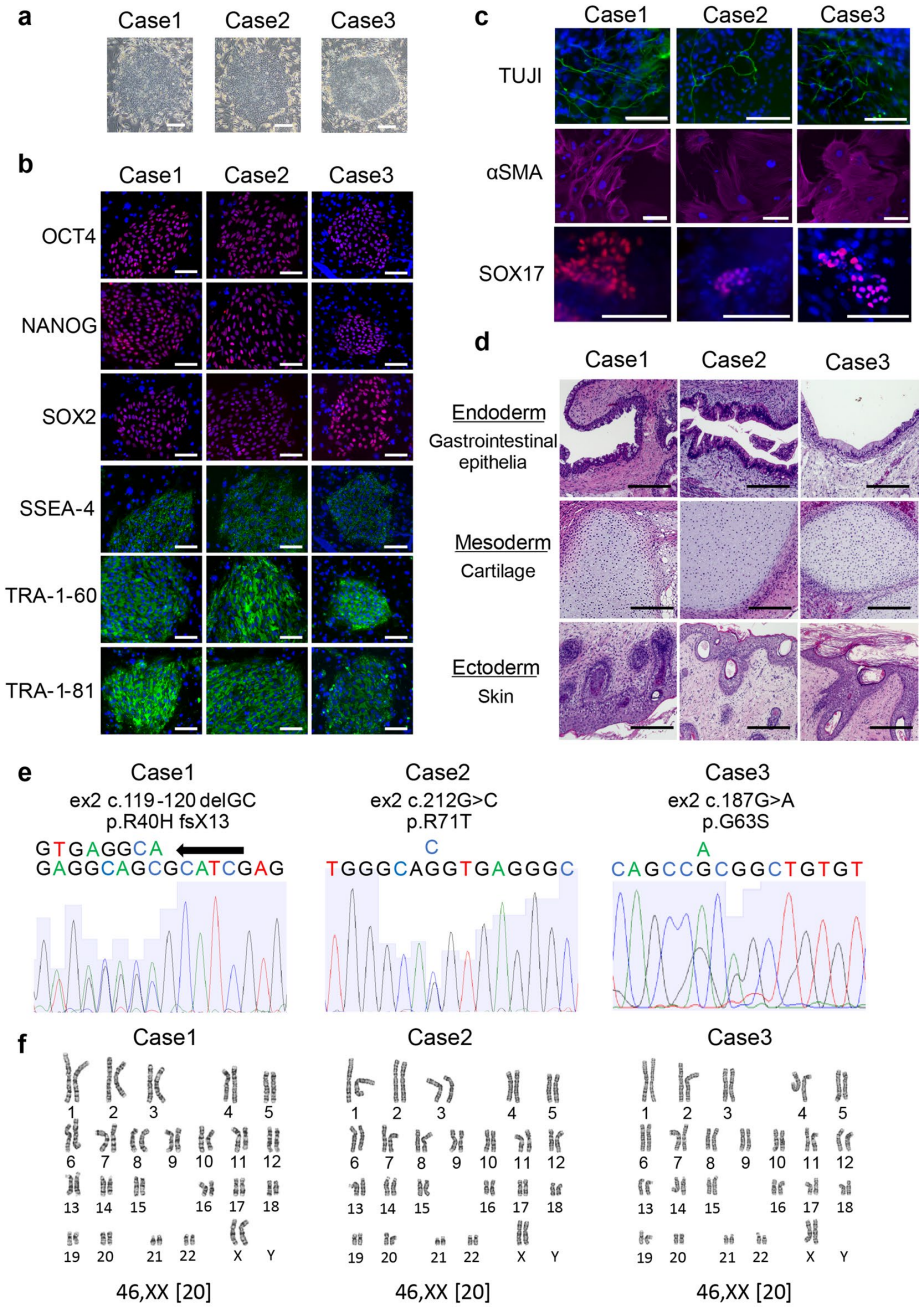
Establishment of induced pluripotent stem cells (iPSCs) from three patients with renal coloboma syndrome (RCS). (**a**) Representative morphology of iPSCs from patients with RCSs. The scale bars represent 100 μm. (**b**) Immunocytochemical analysis of pluripotent markers, OCT4, NANOG, SOX2, SSEA4, TRA-1–60, and TRA-1–81. The scale bars represent 100 μm. (**c**) After 16 days of differentiation of iPSCs with EB formation, the cells were stained with anti-TUJI (ectoderm), αSMA (mesoderm), and SOX17 (endoderm) antibodies. Scale bars represent 100 μm. (**d**) RCS-iPSCs transplanted into immunocompromised nonobese diabetic/severe combined immunodeficient (NOD-SCID) mice developed teratomas containing tissues from three germ layers. These images show the characteristic structure of three germ layers in the teratoma. The scale bars represent 200 μm. (**e**) Representative Sanger sequencing analysis of the mutations in the *PAX2* gene using RCS-iPSCs. Identical mutations to those observed in patients were confirmed in all iPSC clones. (**f**) Normal karyotype by G-banding of RCS-iPSCs.

All iPSC clones from the three patients exhibited characteristic human embryonic stem cell morphology (Fig. 1a) and expressed pluripotency markers, including OCT4, NANOG, SOX2, SSEA4, TRA-1–60, and TRA-1–81 (Fig. 1b). Patient iPSCs could be differentiated into all three germ layers as assessed by both embryoid body (EB) formation, when the cells were cultured in vitro, and teratoma formation, following intratesticular injection of undifferentiated iPSCs into NOD-SCID mice (Fig. 1c,d). The patient iPSCs had the expected heterozygous mutations in the *PAX2* gene (Fig. 1e) and exhibited normal karyotypes (Fig. 1f).

### Induction of kidney lineage cells in control and RCS-iPSCs

**Figure 2:**
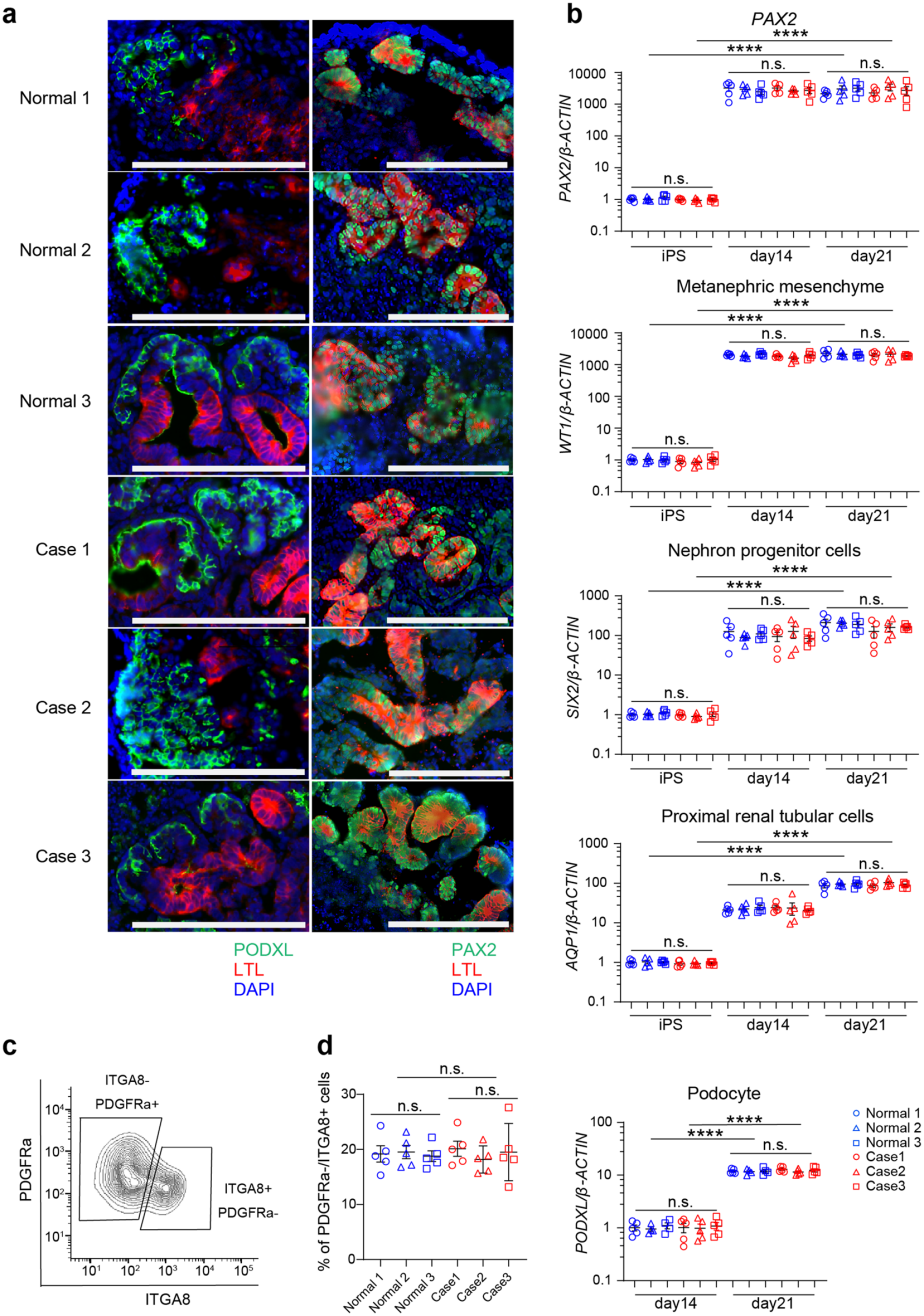
Induction of kidney lineage cells from control and renal coloboma syndrome (RCS) patient-derived induced pluripotent stem cells (iPSCs). (**a**) Control and RCS-iPSCs were differentiated into proximal tubule-like cells (PAX2+ cells, *Lotus tetragonolobus* lectin [LTL]+ cells) and podocyte-like cells (PODXL+) on day 21. The scale bars represent 200 μm. (**b**) Gene expression of kidney lineage markers was equivalent between control and RCS-iPSCs. Data are expressed as the mean ± SEM, n = 5 per group. (**c**) Evaluation of nephron progenitor markers (INTEGRINα8+ PDGFRα−) by fluorescence-activated cell sorting on day 14. (**d**) The induction efficiencies of INTEGRINα8+ PDGFRα− cells were equivalent between control and RCS-iPSCs. Data are expressed as the mean ± SEM, n = 5 per group. Comparison of gene expressions before and after induction was evaluated using Student’s t-test. Comparison of gene expressions between healthy control and RCS samples on each time point were evaluated using Student’s t-test. *Statistically significant, ****P < 0.0001. *n.s.* not significant.

Kidney lineage cells were induced from both control and RCS-iPSCs using previously reported methods^9^. We analyzed the markers of nephron progenitor cells on day 14 and markers of podocyte and proximal tubular cells on day 21, following a previous study^9^. On day 21 of induction, all control and patient iPSC cultures produced cells that were differentially positive by immunohistochemistry for PAX2 and the proximal tubule cell marker LTL (*Lotus tetragonolobus* lectin) and podocyte marker PODXL (podocalyxin) (Fig. 2a). This was confirmed at the RNA level for *PAX2* and *PODXL* and extended to additional kidney lineage markers *WT1*, *SIX2*, and *AQP1* (Fig. 2b). On day 14, the inductive efficiency for generating nephron progenitors, evaluated using the known surface marker INTEGRINα8+ PDGFRα− by fluorescence-activated cell sorting (FACS) (Fig. 2c), was approximately 20% and equivalent in control and patient iPSCs (Fig. 2d). Thus, at the level of light microscopy and marker gene expression, we observed no abnormalities conferred by PAX2 mutation in human nephron development in this model system.

### Identification of PAX2-regulated genes in nephron progenitor cells using RCS-iPSCs and CAGE

**Figure 3:**
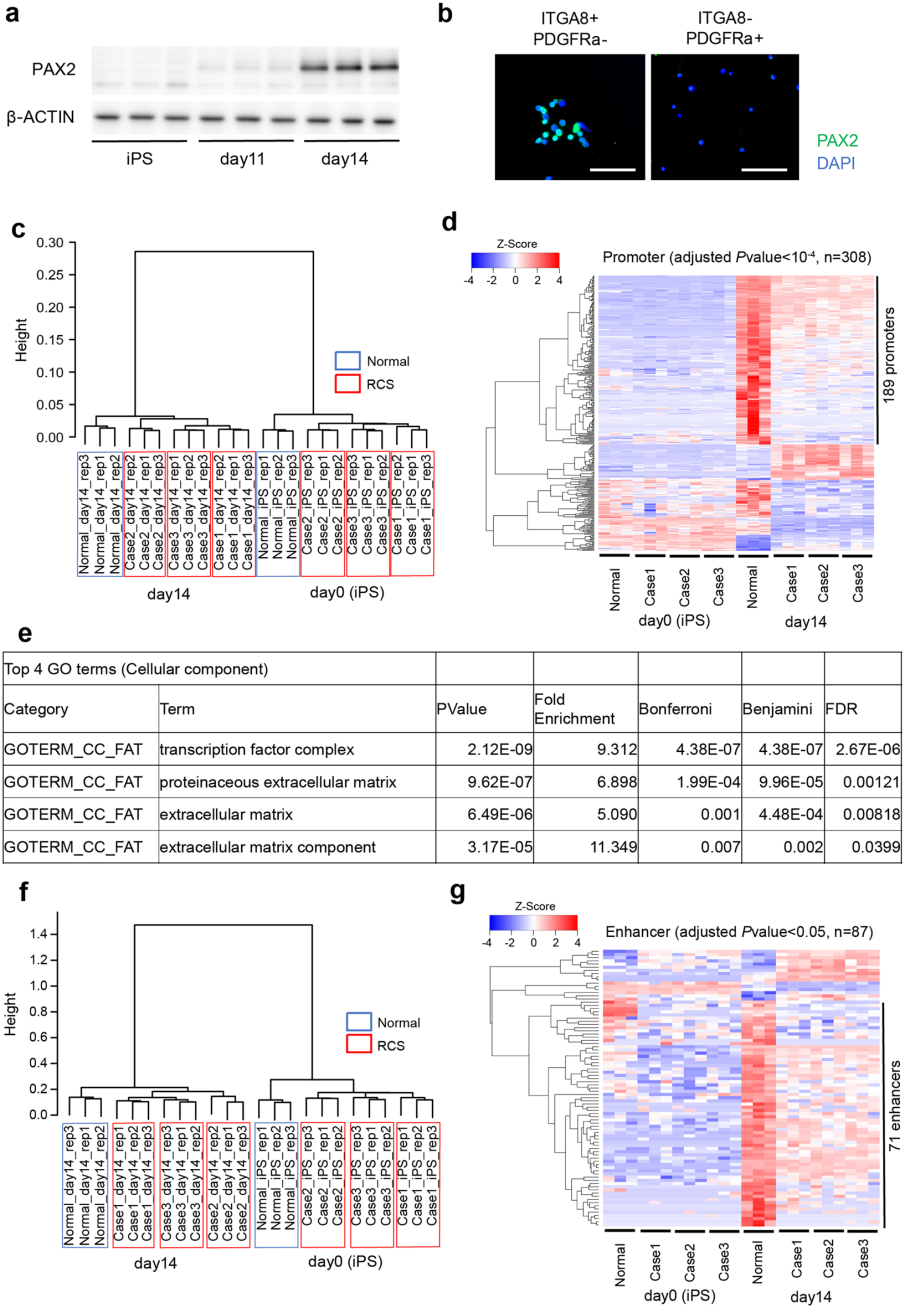
Cap analysis of gene expression (CAGE) analysis during induction to kidney lineage cells. (**a**) Western blots for PAX2 and β-ACTIN during induction from iPSCs to nephron progenitor cells. (**b**) Immunocytochemistry of PAX2 expression in each indicated sorting sample from control iPSCs. The scale bars represent 100 μm. (**c**) Promoter usage profiles of differentiated nephron progenitor cells derived from control and RCS-iPSCs were evaluated using cluster analysis at the indicated time points of culture. Each donor sample was tested in triplicate at each time point. (**d**) Promoter usage profiles of differentiated nephron progenitor cells derived from control and RCS-iPSCs cultured for 14 days were evaluated using heat map analysis. A total of 189 promoters that are differentially activated by the healthy donor compared to patients are arrayed and clustered according to the activation pattern as shown by the dendrogram on the left. (**e**) Results of gene ontology and pathway analysis by The Database for Annotation, Visualization, and Integrated Discovery. The list identifies functional annotations of a subset of differentially expressed genes. (**f**) Enhancer usage profiles of differentiated nephron progenitor cells derived from control and RCS-iPSCs were evaluated using cluster analysis at the indicated time points of culture. (**g**) Enhancer usage profiles of differentiated nephron progenitor cells derived from control and RCS-iPSCs cultured for 14 days were evaluated using heat map analysis.

To identify genes that might be important in human kidney development, we examined the influence of PAX2 expression and RCS *PAX2* mutations on global gene expression in developing nephrons grown from RCS-iPSCs in vitro using CAGE. In our nephron induction protocol, PAX2 was strongly expressed in control cells on day 14 according to both quantitative reverse transcription-polymerase chain reaction (qRT-PCR) and Western blot analysis (Figs. 2b, 3a, Supplementary Fig. S1). Therefore, we selected day 14 as the time point to evaluate the influence of PAX2 in this study. We purified PAX2-positive cells on day 14 of the cultures by sorting nephron progenitor cells using the surface markers INTEGRINα8+ PDGFRα−. By qRT-PCR and immunocytochemistry, we verified that PAX2 expression was higher in this population than in other populations in the culture (Fig. 3b, Supplementary Fig. S1). 110,659 promoters and 36,374 enhancers were identified in CAGE analysis (Supplementary Table S1, S2). As expected, in cluster analysis using these CAGE data, both promoter and enhancer usage data were clearly divided into two primary clusters, one for the iPSCs and the other for INTEGRINα8+ PDGFRα− nephron progenitor cells after 14 days of culture. Both primary clusters were composed of four subclusters, one for the healthy control and one for each of the three patients (Fig. 3c,f). Heat map analysis based on the promoter usage patterns showed that 189 promoters were strongly activated in the control cells compared to those in the patient cells (Fig. 3d, Supplementary Table S3). Gene Ontology (GO) analysis by The Database for Annotation, Visualization, and Integrated Discovery (DAVID) was used to classify these 189 candidate promoters by functional annotation, which revealed 14 transcription factors, 12 extracellular matrix protein genes, 13 extracellular matrix genes, and 7 extracellular matrix component genes (Fig. 3e). Furthermore, heat map analysis based on the enhancer usage patterns showed that 71 enhancers were activated in the control cells compared to those in the patient cells (Fig. 3g).

**Figure 4:**
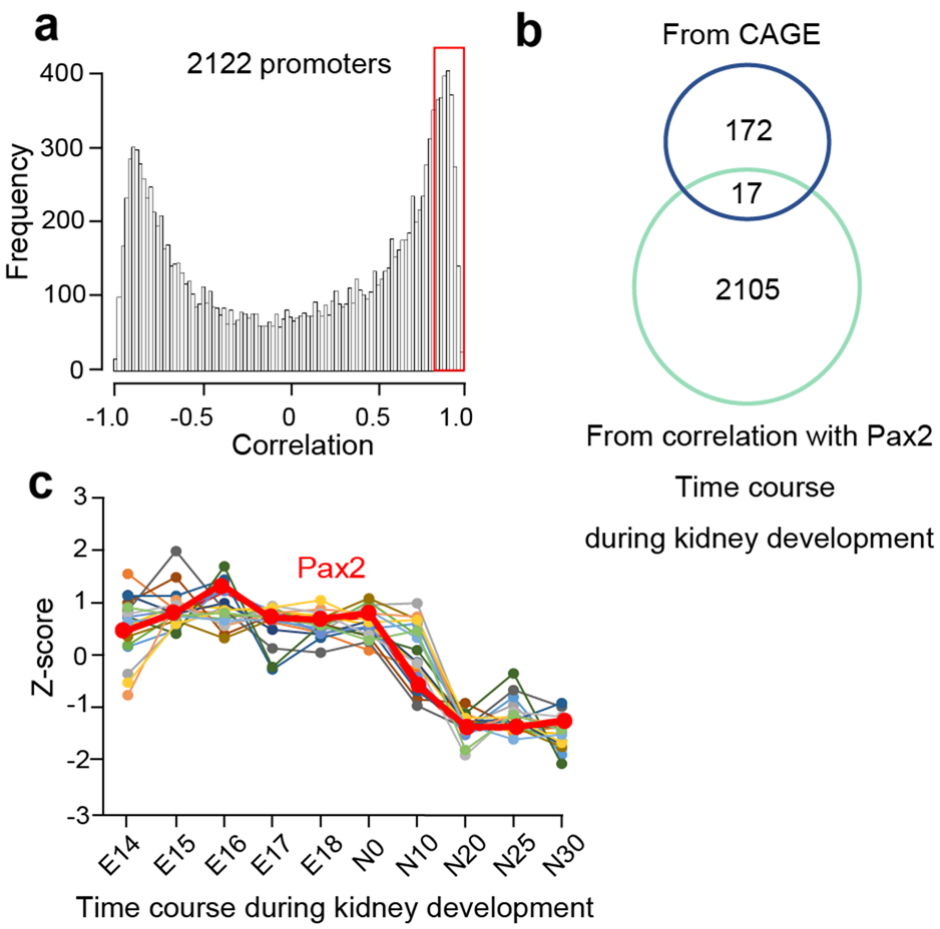
Identification of candidate *PAX2*-regulated kidney development genes by merging the gene list from CAGE and FANTOM database. (**a**) Histogram of correlation with *Pax2*. The histogram indicates the number of promoters based on correlation with Pax2 using global promoter activities during mouse kidney development from the FANTOM5 database. (**b**) Venn diagram shows the method of prioritizing 17 candidate kidney development genes by comparing gene lists identified from human iPSC culture and data mining of PAX2-regulated mouse genes in the FANTOM database. (**c**) Expression of 17 candidate kidney development genes during mouse embryonic kidney development in the FANTOM database. The bold red line represents Pax2. The vertical axis represents the Z-score representing the expression level for each gene.

To identify the best candidates, we focused on 189 activated promoters and not the candidate enhancers because the Functional Annotation of the Mouse/Mammalian Genome (FANTOM) database is best suited for analyzing promoter usage. We searched the FANTOM database for the time course of promoter usage during mouse embryonic kidney development and found 2,122 promoters whose expression correlated with *Pax2* expression levels with correlation coefficients > 0.80 (Fig. 4a). Of these, 17 genes were prioritized for validation as *PAX2*-dependent kidney development genes by merging the gene list established by CAGE for the human nephron culture system and gene list identified by the mouse FANTOM database (Fig. 4b,c). The expression patterns of 17 extracted genes (28 promoters) were similar to that of PAX2 promoter in CAGE data using our human organoid (Supplementary Fig. S2).

### Validation of candidate PAX2-dependent genes involved in renal development

**Figure 5:**
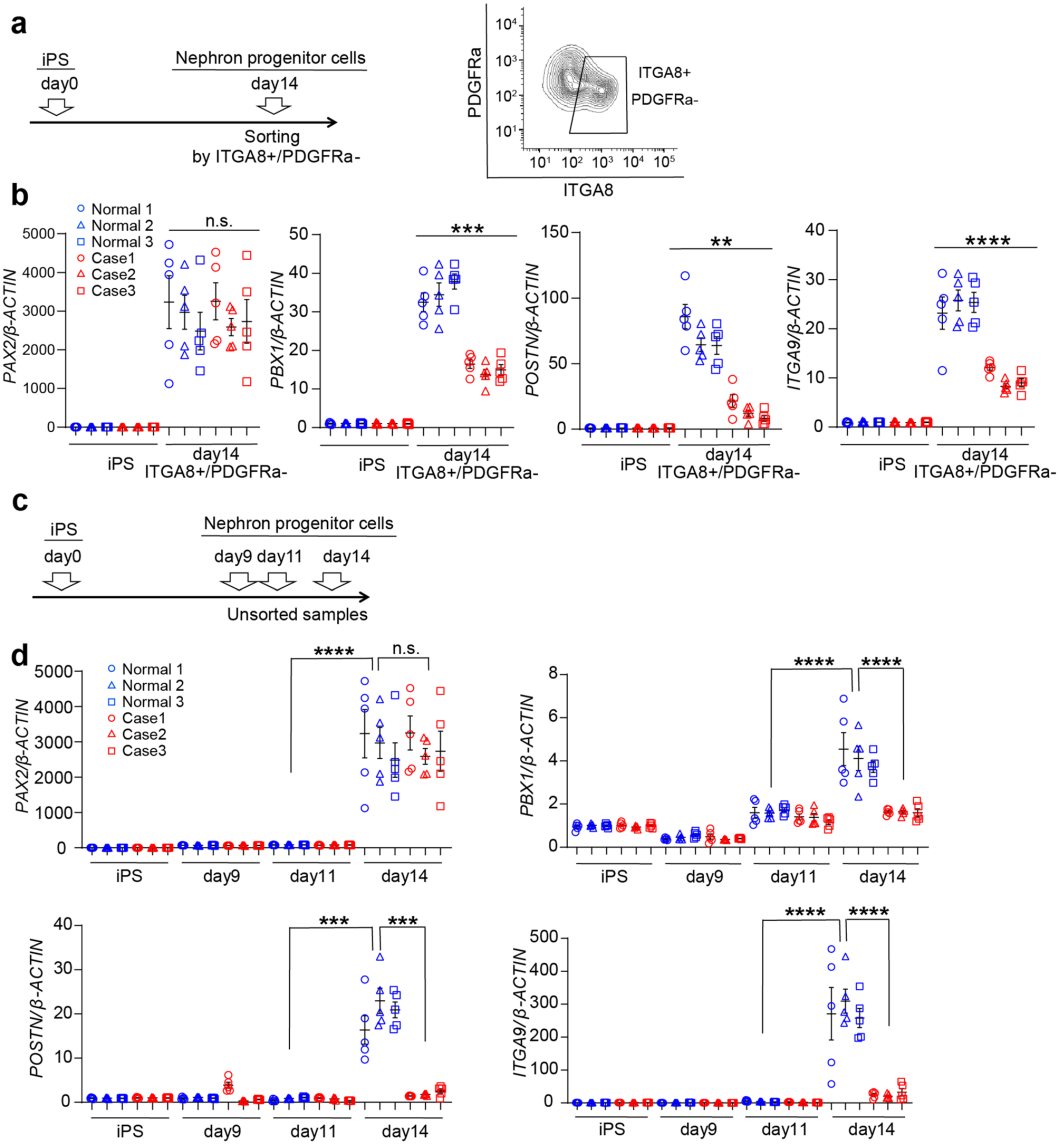
Identification of candidate *PAX2*-regulated kidney development genes by quantitative reverse transcription-polymerase chain reaction (qRT-PCR). (**a**) mRNA samples were collected on iPSCs and sorting nephron progenitor cells using the surface markers INTEGRINα8+ PDGFRα−. (**b**) mRNA expression levels of *PAX2* and candidate genes on iPSCs and sorted nephron progenitor cells using the surface markers INTEGRINα8+ PDGFRα−. (**c**) mRNA samples were collected on iPSCs, day 9, day 11, and day 14. (**d**) mRNA expression levels of *PAX2* and candidate genes during induction of control and RCS-iPSCs to nephron progenitor cells in vitro. Data are expressed as mean ± SEM, n = 5 per group. Comparison of gene expressions between days 11 and 14 in healthy control samples was evaluated using Student’s t-test. Comparison of gene expressions between healthy control and RCS samples on day 14 was evaluated using Student’s t-test. *Statistically significant, **P < 0.01; ***P < 0.001; ****P < 0.0001. *n.s.* not significant.

**Figure 6:**
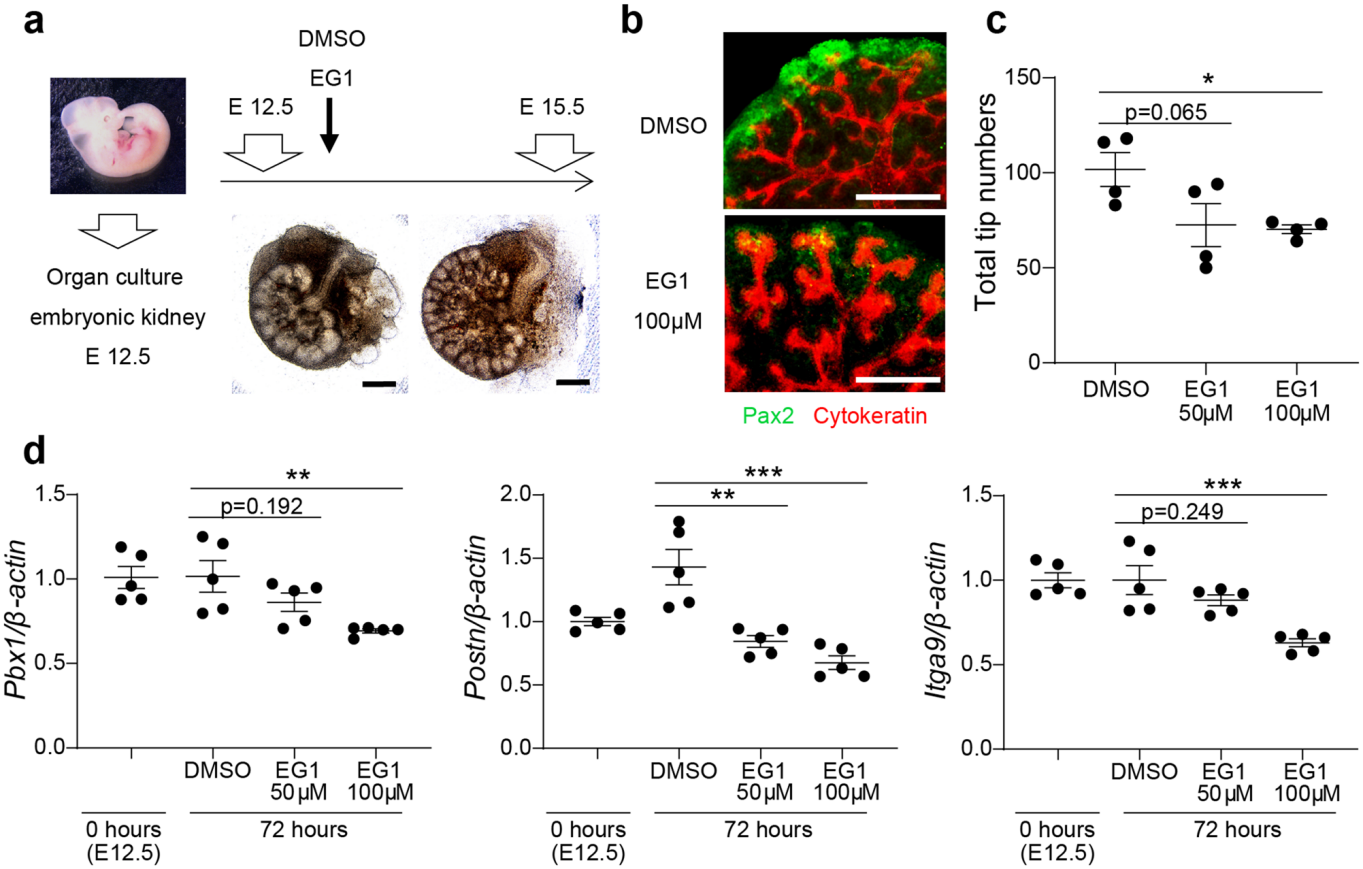
Verification of candidate *PAX2*-regulated kidney development genes in a mouse model. (**a**) Protocol for organ culture using mouse embryonic kidney. Scale bars represent 300 μm. (**b**) Immunocytochemistry of organ culture using mouse embryonic kidney. Scale bars represent 300 μm. (**c**) Total numbers of ureteric tips in organ culture using mouse embryo kidney. Data are expressed as mean ± SEM, n = 4 per group. One-way ANOVA with post hoc Dunnett’s multiple comparisons tests, *P < 0.05. (**d**) mRNA expression levels of candidate *PAX2*-regulated kidney development genes in organ culture using mouse embryonic kidney by quantitative reverse transcription-polymerase chain reaction (qRT-PCR). Data are expressed as mean ± SEM, n = 5 per group. One-way ANOVA with post hoc Dunnett’s multiple comparisons test, **P < 0.01; ***P < 0.001. EG1, PAX2 inhibitor.

To test whether 17 candidate genes were indeed regulated by *PAX2*, expression was re-evaluated by qRT-PCR in two ways: (1) over time in culture for nephrons developing from iPSCs (Fig. 5) and (2) at different times of in vitro culture for mouse embryonic kidney samples (Fig. 6). In the first approach, we verified that the expression of 14 of 17 genes was attenuated at day 14 of culture in patient INTEGRINα8+ PDGFRα− cells compared to those of healthy controls (Fig. 5a,b, Supplementary Fig. S3). Furthermore, we confirmed the data for unsorted nephron progenitor cell clusters over time in culture for nephrons developing from iPSCs. Detailed inspection of the time course revealed that the gene expression patterns for eight candidates (*PBX1*, *POSTN, ITGA9*, *LRRC17*, *HAND2*, *STAR*, *MDK*, and *MEIS1*) most closely resembled the *PAX2* expression pattern, which is strongly induced between days 11 and 14 of culture in the system and downregulated in RCS-iPSC-derived samples (Fig. 5c,d, Supplementary Fig. S4).

Consistent with this, when mouse embryonic kidney cells were cultured in vitro with the Pax2 inhibitor EG1, we observed a reduction at 72 h of incubation in both the number of ureteric tip branches (Fig. 6a–c) and expression of *Pbx1*, *Postn*, and *Itga9*. In this way, we narrowed down 17 candidate genes based on the expression pattern during the differentiation from iPSCs to day 14 and expression pattern of organ culture using mouse embryonic kidney with Pax2 inhibitor. Thereby, we identified the three corresponding genes (*PBX1*, *POSTN*, and *ITGA9*) as the best *PAX2*-regulated target gene candidates for the regulation of kidney development (Fig. 6d, Supplementary Fig. S5).

Finally, we performed chromatin immunoprecipitation (ChIP)-PCR using differentiated nephron progenitor cells to evaluate whether PAX2 binds to the promoters of three genes (*PBX1*, *POSTN*, and *ITGA9*). ChIP-PCR showed that PAX2 may bind to the promoter regions of these genes (Supplementary Fig. S6).

## Discussion

In the present study, we investigated PAX2-regulated genes in human kidney development. We performed sequential and comprehensive analysis using induced kidney lineage cells from RCS-iPSCs with *PAX2* mutations. A total of 189 promoters and 71 enhancers derived from our human-based screening method were identified as PAX2-regulated regions. These data would be useful in human kidney development with respect to human-derived samples. To identify the best candidate genes, we narrowed down the 189 candidate promoters using bioinformatic analysis of a mouse database and model of kidney development. Finally, we identified three top genes (*PBX1*, *POSTN,* and *ITGA9*) that are regulated by *PAX2* and conserved in humans and mice.

Pbx1 is expressed in the nephrogenic mesenchyme during mouse kidney development. *Pbx1* knockout mice have hypoplastic kidneys, poorly defined cortical and medullary regions, and reduced number of differentiating nephrons because of defects in ureteric branching and abnormal expansion of induced mesenchyme^15^. Consistent with this, *PBX1* is one of the causative genes of congenital abnormality of kidney and urinary tract in humans^16,17^. Particularly, *PBX1* mutations cause bilateral renal hypoplasia and unilateral renal agenesis and deafness and developmental delay^16^. These phenotypes are similar to those of patients with RCS with *PAX2* mutations, approximately 10% of whom also have sensorineural hearing loss^18^. Postn is a secreted extracellular matrix protein that is expressed in the renal stroma and ureteric mesenchyme during kidney development. Although Postn is not normally detected in the adult kidney, it is highly induced in renal disease, and its expression is associated with the development or protection of renal lesions^19,20^. Itga9 is known to be involved in cell adhesion. Although its role during kidney development is unclear^21^, it is possible that it is regulated by *Pax2* and involved in kidney development via cell adhesion and cell-to-cell interactions.

Studies on kidney development have been mainly conducted using mouse models. However, there are differences in gene activity between the mouse and human kidneys during early development^22,23^. For example, nephron and interstitial markers mix and persist into epithelializing nephrons in the developing human kidney, unlike the mouse kidney. This is a critical problem especially for the investigation of Pax2 because Pax2 is involved in regulating the border between nephron and interstitial progenitors in the mouse^24^. Human iPSCs are useful in solving this problem. Previous studies have evaluated iPSC organoids targeting some genes as candidates (e.g., *PKD1*, *PKD2,* and *PAX2*) and confirmed that iPSC organoids could reproduce their phenotype^25,26^. In the present study, we evaluated PAX2-regulated genes during human kidney development, especially nephron formation, using RCS-iPSCs and CAGE. Future work will be needed to further interrogate the importance of genes we have prioritized for roles in human kidney development. The general developmental model or bioinformatic pipeline we have described may be useful in obtaining additional clues for the analysis of human kidney development.

The knowledge of PAX2-regulated genes in this study will be useful to examine the kidney development process and other pathophysiologies involving PAX2. RCS is an autosomal dominant disease. Approximately half of patients with RCS have *PAX2* mutations. The causative genes in the remaining half of patients with RCS have not been identified. We previously reported that *KIF26B* is a novel causative gene in patients with RCS who lack *Pax2* mutations by analyzing known genes related to kidney development^14^. The identified candidate PAX2-regulated genes need to be evaluated further to unravel potential disease-causing roles in RCS. We have also reported that patients with *PAX2* mutation-positive RCS have more severe symptoms^14^, but the underlying mechanisms have not been defined. Pax2 is also reactivated in proximal tubular cells after kidney injury^14^. Part of the process of kidney regeneration is similar to the developmental stages of renal tubule specification. Although the relationship between kidney development and repair after kidney injury remains controversial^27^, Pax2 also potentially plays a role in kidney repair and regeneration, as it does during kidney development^28^. Therefore, our study may add helpful knowledge to research on the mechanism of RCS and kidney regeneration via PAX2.

Our investigation has some limitations. First, this study focused on nephron progenitor lineages. However, PAX2 is expressed in both nephron progenitor cells and ureteric buds during kidney development. The role of the *PAX2* gene in ureteric bud development was not evaluated. Recently, some new methods of inducing ureteric buds have been reported^26,29^. Further investigation focusing on ureteric buds will be required to evaluate the role of *PAX2* comprehensively. Second, we alternatively extracted PAX2-positive cells using the surface marker phenotype INTEGRINα8+ PDGFRα− for nephron progenitor cells because the PAX2 antibody for FACS did not work well^30^. The establishment of PAX2 reporter lines by genome editing or single-cell RNA-seq analysis can solve these limitations^31,32^. Third, we couldn’t evaluate the transcriptional activity using PAX2 mutation knock-in model except for RCS-iPSCs. We showed the influence of *PAX2* mutation to the transcriptional activity based on the transcriptional change using CAGE, ChIP-qPCR, and qPCR alternatively. In addition, our previous report provide us the possibility that PAX2 mutations influence transcriptional activity in the view of their structure^14^. Lastly, iPSC clones differ in their inductive efficiency for various reasons^33^. To reduce the effect of this limitation, we selectively used iPSC clones with the highest induction efficiency of OSR1, which is known as the intermediate mesoderm marker.

In conclusion, we successfully established disease-specific iPSCs from patients with RCS and differentiated them into kidney lineage cells. We identified 189 candidate promoters, 71 candidate enhancers, and 3 conservative genes (*PBX1*, *POSTN,* and *ITGA9*) in humans and mice as *PAX2*-regulated targets using patient-derived iPSCs and CAGE. These findings can be used to focus future studies on the mechanism of RCS, kidney development, and kidney regeneration. Further human and animal studies will be required to define the mechanism of the pathogenesis of RCS.

## Methods

### Ethics statement

This study was approved by the Ethics Committees of Kanazawa University and conducted according to the guidelines of the Declaration of Helsinki. Informed consent was obtained from all patients for whom individual information is included in this study in accordance with our institutional research ethics committee (approval number, 2016-046). Animal experiments were approved by the Institute for Experimental Animals, Kanazawa University Advanced Research Center (registration number, AP-143297) and conducted in accordance with the institutional guidelines.

### Generation of iPSCs

RCS-iPSCs were established by transfection of patient PMNCs with episomal vectors encoding *OCT4*, *SOX2*, *KLF4*, *L-MYC*, *LIN28,* and *p53* shRNA as previously described^34^. iPSCs were maintained with feeder-free cultures using StemFit AK02N medium (ReproCELL, Tokyo, Japan) on cell culture plates (CellBIND; Corning, NY, USA) coated with Synthemax (Corning). The medium was changed daily, and passage was performed at 80–90% confluence. iPSCs were routinely monitored for mycoplasma contamination. Three iPSC cell lines (585A1, 585B1, and 648A1), which were generated from PMNCs of 30 s healthy Japanese men using the same method, were used as control iPSCs.

### Mutation analysis

Cell Lysis Solution (Qiagen, Hilden, Germany) and Protein Precipitation Solution (Qiagen) were used according to the manufacturer’s instructions to isolated genomic DNA from RCS-iPSCs. The information of primer pairs used to analyze *PAX2* mutations is provided in Supplementary Table S4.

### Karyotyping

Chromosomal G-band analyses of RCS-iPSCs were performed by Nihon Gene Research Laboratories (Miyagi, Japan).

### EB formation

EB formation was performed as previously described^35^. iPSCs were scraped off, dissociated in primate ES medium, and distributed into low-attachment six-well plates (Corning) for floating culture with EB medium (Knockout Dulbecco’s Modified Eagle Medium, 20% KSR, 0.1 mM non-essential amino acid solution, 2 mM l-glutamine, 500 U/mL P/S, and 0.5 mM 2-mercaptoethanol; all reagents were obtained from Thermo Fisher Scientific). On day 8, the EBs were transferred into gelatin-coated six-well plates and cultured with EB medium for another 8 days.

### Teratoma formation

Teratoma formation was performed as previously described^35^. Undifferentiated iPSCs were transplanted into the testes of 8-week-old male CB17-Prkdcscid/L nonobese diabetic/severe combined immunodeficient (NOD-SCID) mice (Charles River Laboratories Japan, Yokohama, Japan). Nine weeks after transplantation, teratomas were dissected from the mice, fixed in 4% paraformaldehyde (PFA) at 4 ℃ overnight, washed three times with phosphate-buffered solution (PBS), and maintained in 70% ethanol. The fixed teratomas were sectioned and stained with hematoxylin and eosin.

### Differentiation into nephron progenitors and kidney lineage cells

Three RCS-iPSCs and three control human iPS cell lines were induced toward nephron progenitors as previously described^9^, with a minor modification. Briefly, human iPSCs were aggregated at 5 × 10^4^ cells in V-bottomed 96-well low-cell-binding plates (Sumitomo Bakelite Co., Ltd.) using the medium containing 10 μM Y27632 and 0.5 ng/mL human bone morphogenic protein 4 (BMP4, R&D System). After 24 h (on day 1), the medium was changed to one containing 1 ng/mL human activin A (R&D System) and 20 ng/mL human fibroblast growth factor 2 (FGF2, R&D System). On day 3, the medium was changed to one containing 1 ng/mL human BMP4 and 10 μM CHIR99021 (Wako). On day 9, the medium was changed to one containing 10 ng/mL activin A, 3 ng/mL BMP4, 3 μM CHIR99021, 0.1 μM retinoic acid (Sigma), and 10 μM Y27632. On day 11, the medium was changed to one containing 1 μM CHIR99021, 5 ng/mL human FGF9 (R&D System), and 10 μM Y27632. On day 14, induced aggregates were cultured with NIH3T3 fibroblast expressing Wnt4 cells^36^ supplied with DMEM containing 10% fetal bovine serum (FBS).

### Immunocytochemistry

Cells were fixed with 4% PFA at 4 ℃ overnight. Fixed cells were blocked with 0.25% Triton X-100, 3% bovine serum albumin, and 1% normal donkey or goat serum (Millipore, MA, USA) in PBS for 1 h at room temperature. Primary antibodies were diluted in blocking solution and incubated overnight at 4 ℃. The secondary antibodies were incubated for 1 h at room temperature. The secondary antibodies were Alexa 488, 546, 594, or 647 conjugated. Detailed information on antibodies is provided in Supplementary Table S5.

### RNA extraction, reverse transcription, and qRT-PCR

Total RNA was obtained with a High Pure RNA Isolation Kit (Roche, Basel, Switzerland), and cDNA was synthesized with a High-Capacity RNA-to-cDNA Kit (Applied Biosystems, CA, USA). SYBR Green PCR Master Mix (Bio-Rad, CA, USA) was used for qRT-PCR. Reactions were performed following the manufacturer’s protocol (ViiA 7 real-time PCR system, Applied Biosystems) and analyzed using the ΔΔCt method. β-ACTB for humans and mice were used as housekeeping genes. Detailed information on primers is provided in Supplementary Table S4.

### FACS

Induced cell aggregates from iPSCs on day 14 were dissociated and blocked using PBS containing 10% FBS. Staining of cell surface markers was performed in PBS containing 2% FBS for 30 min on ice. The stained cells were analyzed and sorted (FACSAria II cell sorter, BD Biosciences, CA, USA). The sorted cells were analyzed by qRT-PCR, immunocytochemistry, and CAGE. Detailed information on antibodies is provided in Supplementary Table S5.

### Western blot analysis

Samples were solubilized in 1× RIPA Lysis Buffer (Merck Millipore, Billerica, MA, USA) supplemented with phosphatase inhibitor and protease inhibitor (Roche) and analyzed by SDS–polyacrylamide gel electrophoresis, followed by immunoblotting. The analysis of blotted membranes was conducted using ImageJ software (National Institutes of Health, Rockville, MD, USA). Detailed information on antibodies is provided in Supplementary Table S5.

### CAGE library preparation and sequencing

CAGE libraries were generated from total RNA according to the previously described no-amplification non-tagging CAGE protocol^37^. Briefly, cDNAs were reverse transcribed from total RNA using random primers. Next, 5′ caps of RNAs were biotinylated. After RNase I digestion of single-stranded RNAs, biotinylated RNA/cDNA molecules were captured by streptavidin-coated magnetic beads. Subsequently, single-stranded cDNAs were released from hybrid RNA/cDNA molecules, and adapters were ligated to single-stranded cDNAs. Double-stranded cDNAs were obtained by second strand synthesis. CAGE libraries were sequenced on an Illumina HiSeq 2500 platform in single-read mode.

### Alignment of CAGE data

MOIRAI pipeline (http://fantom.gsc.riken.jp/software/)^38^ was used for the following preprocessing before mapping. Sequenced reads were split by barcode, and barcode sequences were trimmed. Reads with base “N” and reads mapped to ribosomal RNA (human: GenBank U13369.1) were removed. Reads were aligned to human genome hg19 using STAR version 2.5.0a^39^ with the following parameters: –outSAMtype BAM SortedByCoordinate–outFilterMultimapNmax 1. The index for STAR was generated based on default settings, except for information on splice junctions obtained from Gencode v27lift37 (“comprehensive”)^40^.

### Expression analysis of CAGE data

Annotation files for human FANTOM5 promoters^41^ were downloaded from http://fantom.gsc.riken.jp/5/datafiles/latest/extra/CAGE_peaks/. Coverage of 5′ positions at the single-base level was calculated using bedtools v2.25.0^42^ in a strand-specific manner with the following parameters: genomecov -5 -bg -strand + or genomecov -5 -bg -strand -. bedGraph files were converted to bigWig files using bedGraphtobigWig^43^. Reads mapped to FANTOM5 promoters were counted using bigWigAverageOverBed^43^. We identified differentially expressed promoters between nephron progenitor cells from RCS-iPSCs (n = 9) and controls (n = 3) cultured for 14 days using edgeR version 3.16.5^44^. Briefly, count data were normalized based on relative log expression, and the dispersion values were estimated by the quantile-adjusted conditional maximum likelihood method. Then, exact tests were computed for differences in the means between two groups of negative-binomially distributed counts. *P* values were adjusted by Benjamini–Hochberg method^45^. For clustering and heatmap analysis, log_2_ [counts per million (CPM) plus one] was utilized. Clustering analysis was performed using identified 110,659 promoters (read counts in at least one sample > 0), and the distance was measured by one minus Pearson’s correlation coefficient. Heatmap analysis of differentially expressed promoters was performed using gplots version 2.2.1^46^. GO analysis of the differentially expressed genes was conducted using DAVID (https://david.ncifcrf.gov/home.jsp)^47,48^. De novo enhancers were identified as previously reported^49^. Identified enhancers were combined with FANTOM-NET enhancers^50^ obtained from http://fantom.gsc.riken.jp/5/suppl/Hirabayashi_et_al_2019/data/Supplementary_Data_1_Human_FANTOM-NET_enhancers.bed.gz. To count reads mapped to the enhancer regions, a similar analysis was performed as for promoters. Then, the count data were normalized using the normalization factors computed by the count data of promoters. After filtering enhancers with no expression in any sample, differential enhancer expression analysis was performed as described above for differential promoter expression analysis. A prior count of 0.25 was added to counts for each enhancer, and log_2_ [CPM] was calculated for clustering and heatmap analysis. Clustering analysis was performed using identified 36,374 enhancers (read counts in at least one sample > 0), and the distance measure was the same as described for the clustering analysis of promoters. The heatmap was generated with differentially expressed enhancers. In this subsection, R version 3.3.3^51^ was used for analyzing and plotting CAGE data. Moreover, CAGE data were analyzed using Z-score after log2 conversion. The correlation to the PAX2 promoter was evaluated. CAGE data were available at the DNA Data Bank of Japan (DDBJ) database (accession number DRA009653).

### Data mining from FANTOM database

The promoter activities during mouse kidney development were obtained using FANTOM5 Table Extraction Tool. The data were analyzed using Z-score after log2 conversion. Finally, the correlation to Pax2 promoter (indicated as p@chr19:44831187. 0.44831204,+) was evaluated.

### Organ culture

C57BL/6J mice were used in this study. Kidney rudiments were microdissected on embryonic day 12.5 (E12.5) and cultured on 0.4-μm Transwell filter inserts (Corning) in DMEM containing 10% FBS under humidified 5% CO_2_/95% air at 37 °C for 3 days. DMSO (Sigma) or increasing concentrations of EG1 (ChemBridge, CA, USA) were used to assess the influence of Pax2.

### ChIP-qPCR

Induced cell aggregates from iPSC at day 14 were dissociated and fixed with fresh 1% formaldehyde (final concentration) for 10 min at room temperature. Simple ChIP Enzymatic Chromatin IP Kit (Cell Signaling Technologies, MA, USA) was used according to the manufacturer’s instructions to perform the ChIP experiment. Chromatin from 5 × 10^6^ cells was used for each ChIP experiment. After chromatin fragmentation by enzyme treatment and sonication, ChIP-qPCR was performed with three technical replicates. ChIP was performed with rabbit anti-Pax, rabbit anti-histone H3 as a technical positive control (Cell Signaling Technologies, MA, USA), and normal rabbit IgG as a negative control (Cell Signaling Technologies, MA, USA). After reverse cross-linking and DNA purification, purified DNA was used for ChIP-qPCR. The reactions of ChIP-qPCR were performed in the same method as that in the qRT-PCR section. Detailed information on primers and antibodies is provided in Supplementary Table S4 and S5.

### Statistical analyses

The results are presented as mean ± SEM. Unpaired two-tailed Student’s t-test was used to compare two groups, and a P-value < 0.05 was considered significant. For multiple group comparisons, one-way ANOVA followed by post hoc correction with Dunnett’s test or Bonferroni’s test was applied. Statistical analyses were performed using GraphPad Prism 8.0.

## Supplementary Information

Supplementary Legends.

Supplementary Figures.

Supplementary Table S1.

Supplementary Table S2.

Supplementary Table S3.

Supplementary Table S4.

Supplementary Table S5.

## Data Availability

The data underlying this study are available in the DDBJ database at https://ddbj.nig.ac.jp/DRASearch/ and can be accessed with DRA009653.
