# Supplementary material for: Identification of candidate PAX2-regulated genes implicated in human kidney development
Source: Sci Rep. 2021 Apr 27;11:9123. doi: 10.1038/s41598-021-88743-1 (PMC8079710; doi:10.1038/s41598-021-88743-1)
Supplement: Supplementary file 1 — Supplementary Legends. [file 41598_2021_88743_MOESM1_ESM.docx]

**Supplementary data**

## **Figure S1. Gene expression of *PAX2* during induction of kidney lineage cells from induced pluripotent stem cells (iPSCs) and sorted samples**

(**a**) Gene expression of *PAX2* during induction from iPSCs to kidney lineage cells. Data are expressed as mean ± SEM, n = 5 per group. (**b**) Gene expression of *PAX2* in each sorted sample. Data are expressed as mean ± SEM, n = 5 per group. One-way ANOVA with post hoc Bonferroni’s test, ****P* < 0.001; *****P* < 0.0001. n.s., not significant. (**c**) Uncropped Western blot images for Figure 3a.

**Figure S2. Association between *PAX2* promoter and candidate *PAX2*-regulated promoters using CAGE data from our normal human organoid samples.**

(**a**)(b) Expression of 17 candidate genes (28 promoters) and some housekeeping promoters during the differentiation from iPSCs to nephron progenitor cells. The bold red line represents PAX2. The vertical axis represents the Z-score representing the expression level for each gene. (**c**) We examined the correlations of 17 candidate genes (28 promoters) and some housekeeping promoters with PAX2 promoter using CAGE data from our normal human organoid samples.

**Figure S3. mRNA expression levels of candidate genes on iPSCs and sorted nephron progenitor cells using the surface markers INTEGRINα8+ PDGFRα–**

## mRNA expression levels of candidate genes on iPSCs and sorted nephron progenitor cells using the surface markers INTEGRINα8+ PDGFRα–. This figure shows candidate genes that were attenuated at day 14 of culture in patient INTEGRINα8+ PDGFRα–cells compared to the healthy control subject, except for PBX1, POSTN, and ITGA9. Data are expressed as mean ± SEM, n = 5 per group. Comparison of gene expressions between healthy control and RCS samples on day 14 was evaluated Student’s t-test. *Statistically significant, ***P* < 0.01; ****P* < 0.001; *****P* < 0.0001.

**Figure S4. mRNA expression levels of candidate genes during induction of control and RCS-iPSCs to nephron progenitor cells in vitro**

## mRNA expression levels of candidate genes during induction of control and RCS-iPSCs to nephron progenitor cells in vitro. The gene expression patterns for eight candidates (*PBX1*, *POSTN, ITGA9*, *LRRC17*, *HAND2*, *STAR*, *MDK,* and *MEIS1*) most closely resembled the *PAX2* expression pattern, which is strongly induced between days 11 and 14 of culture in the system and downregulated in RCS-iPSC-derived samples. The gene expression data of *PBX1*, *POSTN*, and *ITGA9* are shown in Fig. 5D. Data are expressed as mean ± SEM, n = 5 per group. Comparison of gene expressions between day 11 and 14 in healthy control samples was evaluated using Student’s t-test. Comparison of gene expressions between healthy control and RCS samples on day 14 was evaluated using Student’s t-test. *Statistically significant, ***P* < 0.01; ****P* < 0.001; ****P < 0.0001.

## **Figure S5. Verification of candidate *PAX2*-regulated kidney development genes in a mouse model**

mRNA expression levels of candidate *PAX2*-regulated kidney development genes in organ culture using mouse embryonic kidney by quantitative reverse transcription-polymerase chain reaction (qRT-PCR). Data are expressed as mean ± SEM, n = 5 per group. One-way ANOVA with post hoc Dunnett’s multiple comparisons test. EG1, PAX2 inhibitor.

**Figure S6. Chromatin immunoprecipitation (ChIP)-qPCR for PBX1, POSTN, and ITGA9 using differentiated nephron progenitor cells from healthy human iPSCs**

(**a**) Schematic representation of PBX1, POSTN, and ITGA9 promoter regions. Red squares indicate identified promoters in CAGE analysis. Thick bars indicate target sequences of each primer. Target sequences were decided by referencing some peak closed to target promoters from preliminary ChIP-Seq. (**b**) The expression levels of three genes, compared with input samples by qRT-PCR using ChIP DNA with two PAX2 antibodies　(shown as PAX2 Ab #1 and PAX2 Ab #2), a histone H3 antibody as a technical positive control, and normal IgG as a negative control. ChIP DNA samples were prepared using differentiated nephron progenitor cells from healthy human iPSCs. ChIP-qPCR data was normalized with ‘Percent Input Method’ as described in (<https://www.thermofisher.com/us/en/home/life-science/epigenetics-noncoding-rna-research/chromatin-remodeling/chromatin-immunoprecipitation-chip/chip-analysis.html>). Data are expressed as mean ± SEM, n = 3 per group. Comparison of gene expressions between PAX2 antibody-treated samples, histone H3 antibody-treated samples and normal IgG-treated samples were evaluated using One-way ANOVA with post hoc Dunnett’s multiple comparisons test. *Statistically significant, ***P* < 0.01; ****P* < 0.001; ****P < 0.0001.
