# Supplementary material for: Identification of candidate PAX2-regulated genes implicated in human kidney development
Source: Sci Rep. 2021 Apr 27;11:9123. doi: 10.1038/s41598-021-88743-1 (PMC8079710; doi:10.1038/s41598-021-88743-1)
Supplement: Supplementary file 2 — Supplementary Figures. [file 41598_2021_88743_MOESM2_ESM.pdf]

Identification of candidate PAX2-regulated genes implicated in human kidney development

Yuta Yamamura[1], Kengo Furuichi[2*], Yasuhiro Murakawa[3], Shigeki Hirabayashi[3], Masahito Yoshihara[4], Sako Keisuke[1], Shinji Kitajima[1], Tadashi Toyama[1], Yasunori Iwata[1], Norihiko Sakai[1], Kazuyoshi Hosomichi[5], Philip M. Murphy[6], Atsushi Tajima[5], Keisuke Okita[7], Kenji Osafune[7], Shuichi Kaneko[8], Takashi Wada[1*]

Figure S1

a

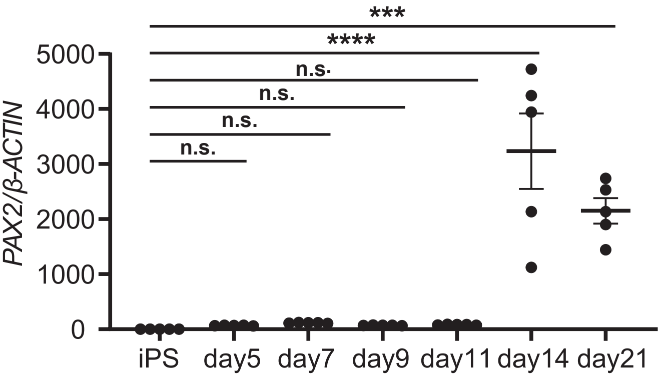

b

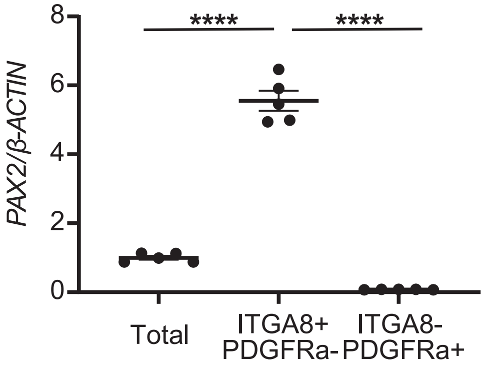

c

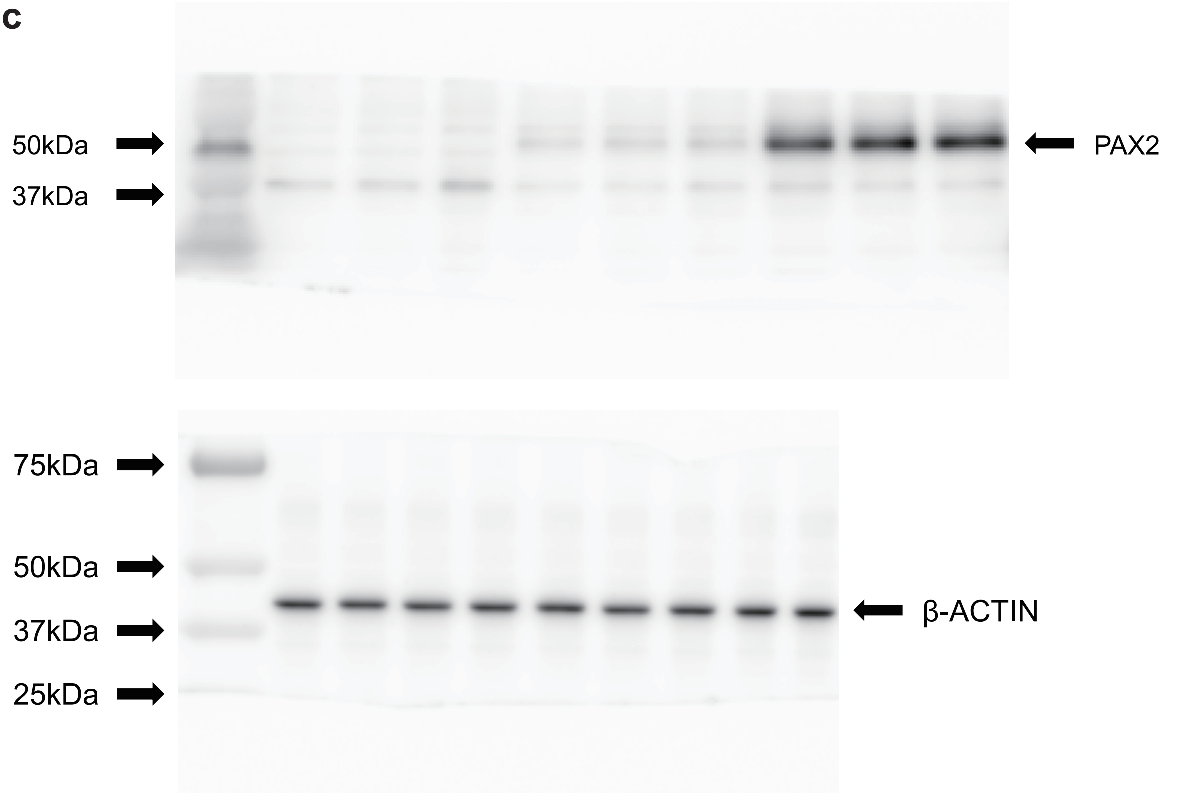

Figure S2

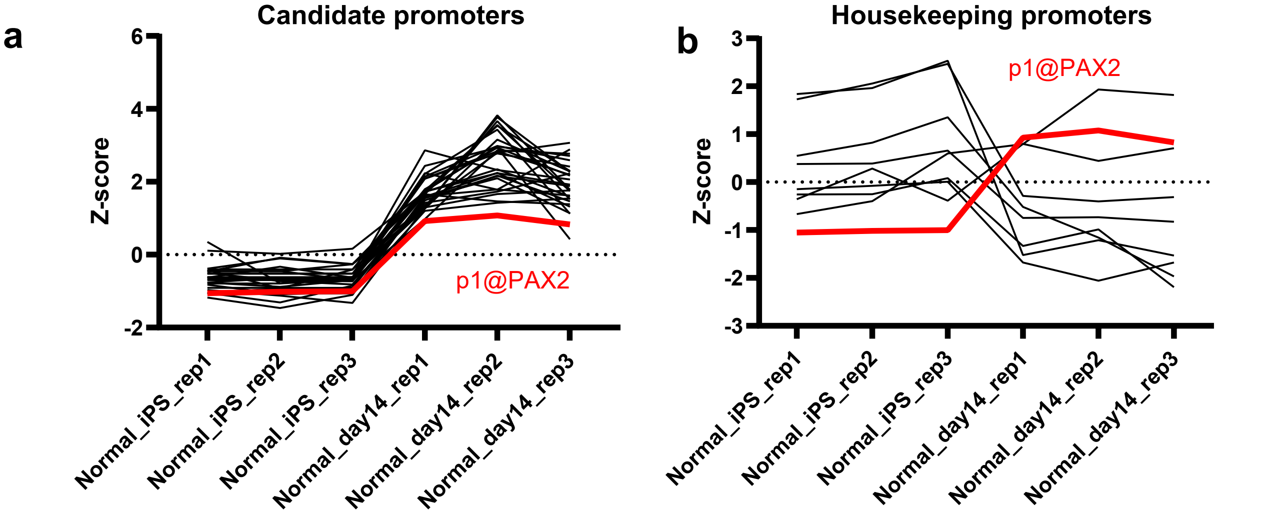

c

	Normal_iPS rep1	Normal_iP Srep2	Normal_iPS rep3	Normal_day14 rep1	Normal_day1 4rep2	Normal_day14 rep3	Correleation
**p1@PAX2**	-1.0540926	-1.019158	-1.0012991	0.926536622	1.079790198	0.828888776	1
**Candidate**							
**p1@PBX1**	-0.7611194	-0.881975	-0.6935628	1.659245668	1.462656731	1.383150393	0.994372806
**p1@POSTN**	-0.8203817	-1.082022	-1.0419486	1.608551612	1.791996907	1.62444934	0.996355661
**p1@ITGA9**	-0.4943334	-0.720961	-0.8141476	1.49099772	2.16855855	1.465254462	0.987908029
**p3@MDK**	-0.6180089	-0.702422	-0.7293544	1.732785774	2.238202103	1.92416115	0.996123938
**p10@MDK**	0.11148479	0.0200866	0.1613892	1.720386985	2.343757393	1.587555845	0.982125625
**p1@SEPT11**	-1.0506944	-0.930094	-0.9932542	1.202438673	1.425836528	1.544002977	0.992147348
**p4@SEPT11**	0.34899488	-0.78629	-0.612406	1.266332431	3.157922479	2.284752981	0.90324767
**p2@GATA3**	-0.6299507	-0.328146	-0.6299507	1.358492466	2.79785855	3.068733583	0.935530859
**p10@MEIS1**	-0.6949071	-0.694907	-0.6949071	2.238077125	1.783113239	2.887680956	0.960043909
**p3@MEIS1**	-0.6330187	-0.633019	-0.6330187	2.86098532	2.324291335	2.066266071	0.987790236
**p2@LRRC17**	-0.9109477	-0.910948	-0.9109477	1.553154479	2.158598231	1.88123777	0.994547265
**p1@SCT**	-0.3839908	-0.101889	-0.2634307	1.318914447	3.666068134	1.515363529	0.880499931
**p1@FAM198B**	-0.6813962	-0.681396	-0.6813962	2.218999021	3.43323563	1.126075252	0.938606911
**p2@COL16A1**	-0.463525	-0.085569	-0.2551115	1.660989594	3.55513911	1.745610637	0.926902472
**p1@HOXD11**	-0.7877123	-0.787712	-0.6769563	2.43570619	2.939277	0.422530798	0.898101748
**p1@TWIST1**	-1.0617683	-1.061768	-1.0617683	1.567878034	2.099959367	1.135558191	0.991080299
**p2@NID2**	-1.0521929	-1.306715	-0.8522499	1.425778671	1.732597726	2.227983994	0.974499059
**p1@NID2**	-0.9690307	-1.122694	-1.3245119	1.299985727	1.665122361	1.763172026	0.987290672
**p2@HAND2**	-0.4008435	-0.400843	-0.4008435	1.291607136	3.772773841	2.267064618	0.912595398
**p3@HAND2**	-0.5217087	-0.521709	-0.5217087	2.140696273	2.983872029	2.588657544	0.991631194
**p1@HAND2**	-0.6670314	-0.667031	-0.6670314	2.086294203	2.776211146	2.415435256	0.994887008
**p1@STAR**	-0.6193423	-0.619342	-0.6193423	1.741495411	2.973268612	2.706932964	0.974880989
**p4@GATA6**	-0.5137729	-0.513773	-0.5137729	1.269100084	3.820968924	1.781223038	0.901433826
**p2@GATA6**	-0.4572226	-0.457223	-0.2634873	1.759795734	3.537463842	2.294176792	0.957044564
**p3@GATA6**	-0.4475206	-0.449938	-0.7051423	0.976275105	2.861566464	2.769614122	0.912797407
**p1@GATA6**	-0.7638938	-0.638187	-0.6657664	1.799713221	2.934315346	2.172444799	0.985276106
**p1@CD248**	-1.1793378	-1.460113	-1.1065039	1.587375116	2.242556016	1.314044622	0.991256427
**p1@MFAP4**	-0.4175394	-0.924064	-0.4077551	2.091077593	2.835689394	2.187809454	0.988236928
**Housekeeping**							
**p1@ACTB**	-0.2587909	-0.253304	0.0847198	-1.331911014	-0.987134389	-2.190777053	-0.839522917
**p1@GAPDH**	1.72834005	2.056469	2.4699494	-0.288805352	-0.404593442	-0.313341981	-0.980524217
**p1@PPIA**	0.37961954	0.389843	0.658403	-0.745503374	-0.733070746	-0.82543422	-0.980816089
**p1@B2M**	-0.6684367	-0.39969	0.5955782	0.793770981	1.933447621	1.819334623	0.854275504
**p1@RPL32**	-0.3563607	0.2824596	-0.3877652	0.799173147	0.440550538	0.705564792	0.840127362
**p1@SDHA**	1.83705785	1.9646534	2.5304308	-1.520204068	-1.214550627	-1.534889084	-0.982597133
**p1@YWHAZ**	0.5488969	0.824831	1.3528388	-0.51942996	-1.159949279	-1.966942131	-0.893641478
**p1@HPRT1**	-0.1451772	-0.076233	0.0085041	-1.679608793	-2.057498954	-1.676041238	-0.994510622

Figure S3

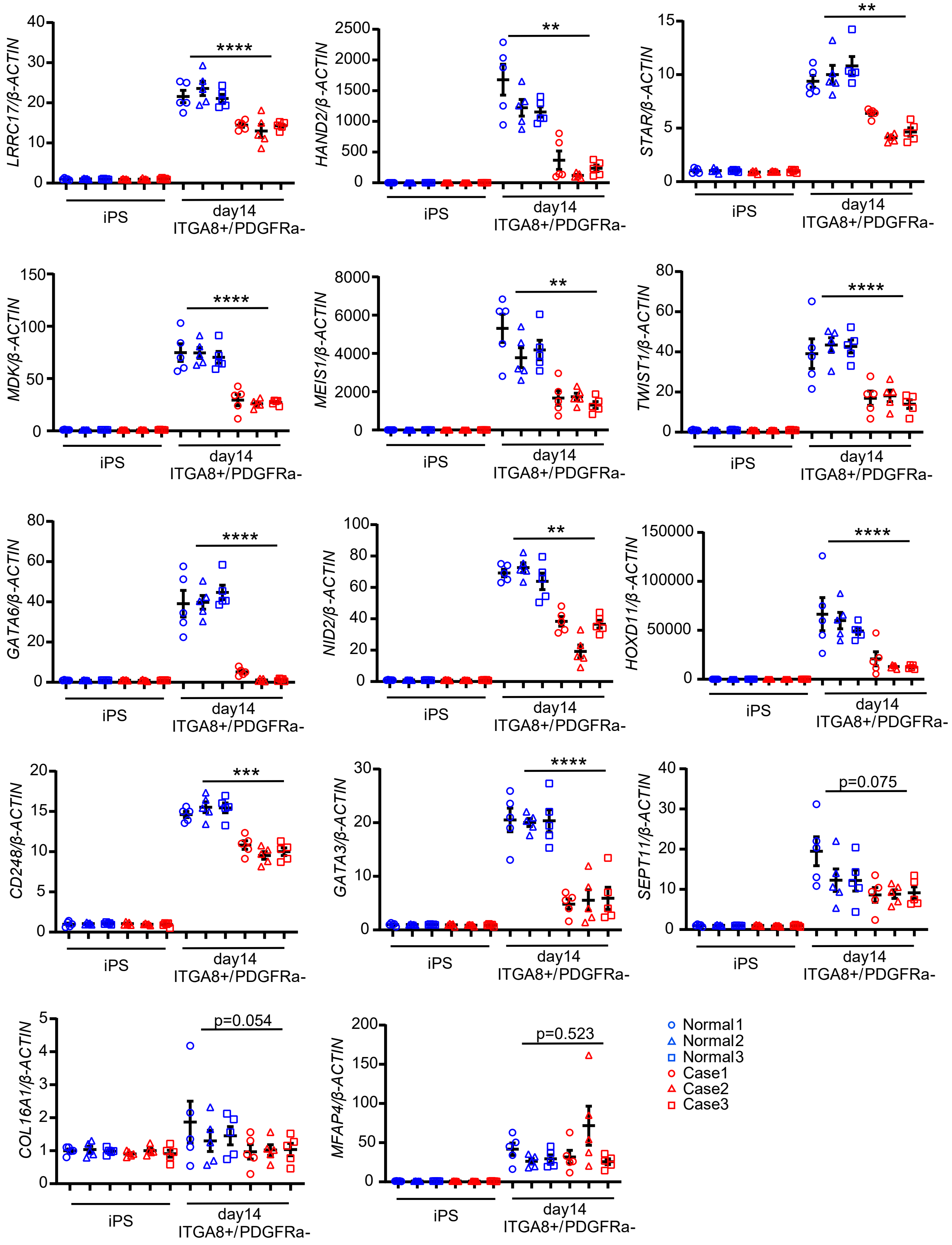
LRRC17/β-ACTIN
HAND2/β-ACTIN
STAR/β-ACTIN
MDK/β-ACTIN
MEIS1/β-ACTIN
TWIST1/β-ACTIN
GATA6/β-ACTIN
NID2/β-ACTIN
HOXD11/β-ACTIN
CD248/β-ACTIN
GATA3/β-ACTIN
SEPT11/β-ACTIN
COL16A1/β-ACTIN
MFAP4/β-ACTIN
****
**
***
p=0.075
p=0.054
p=0.523
iPS
day14
ITGA8+/PDGFRa-
Normal1
Normal2
Normal3
Case1
Case2
Case3

Figure S4

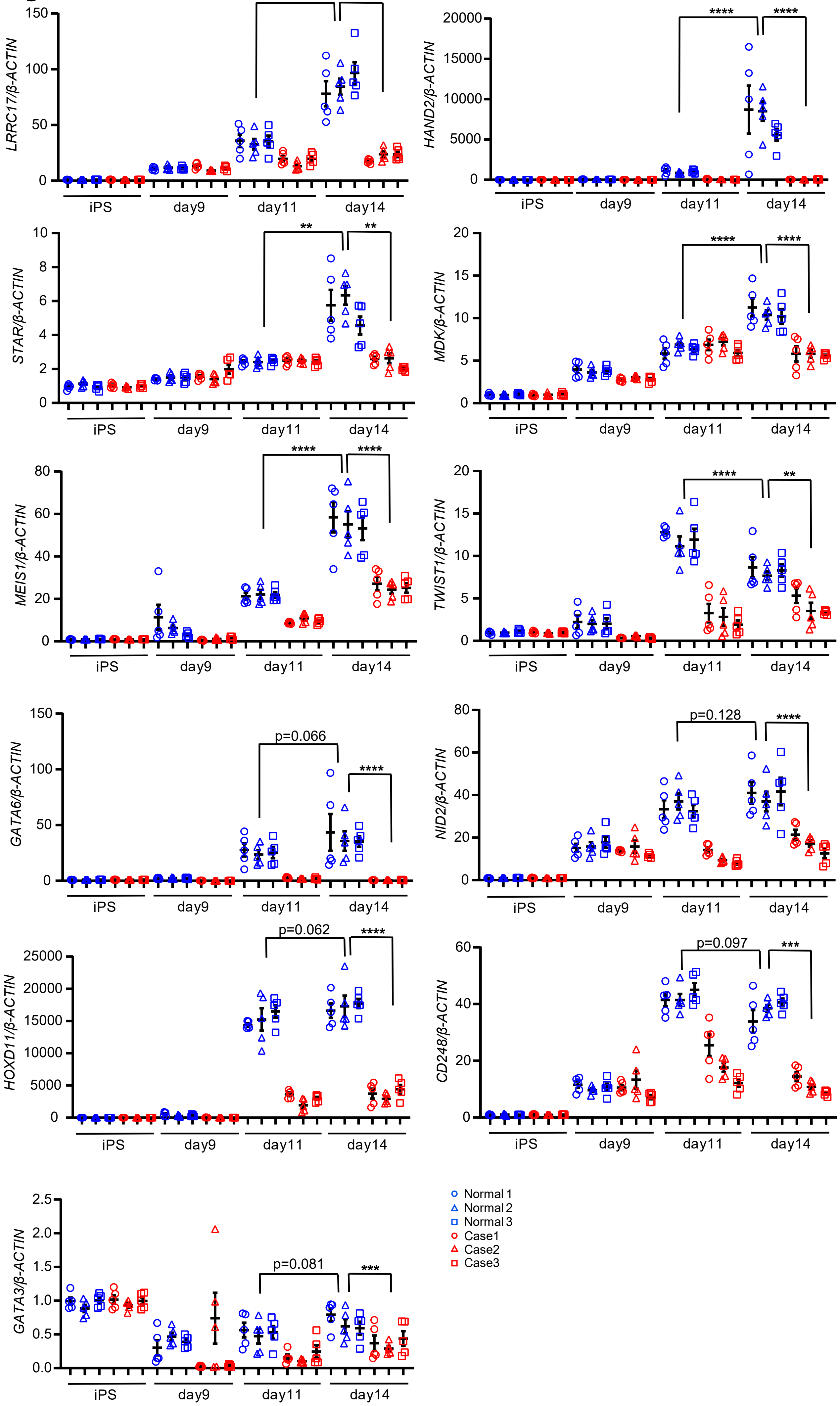
LRRC17/β-ACTIN
HAND2/β-ACTIN
STAR/β-ACTIN
MDK/β-ACTIN
MEIS1/β-ACTIN
TWIST1/β-ACTIN
GATA6/β-ACTIN
NID2/β-ACTIN
HOXD11/β-ACTIN
CD248/β-ACTIN
GATA3/β-ACTIN
iPS
day9
day11
day14
****
***
**
p=0.066
p=0.128
p=0.062
p=0.097
p=0.081
Normal 1
Normal 2
Normal 3
Case1
Case2
Case3

Figure S5

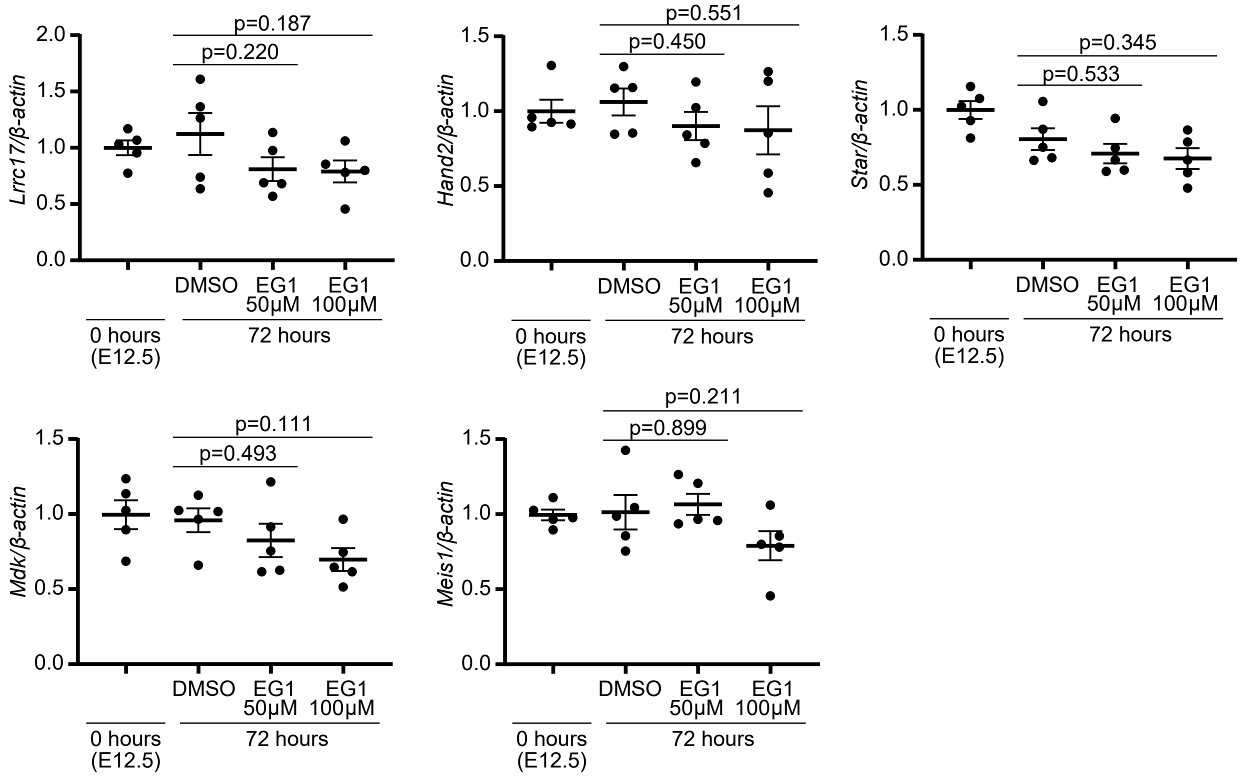

Lrrc17/β-actin
p=0.187
p=0.220
Hand2/β-actin
p=0.551
p=0.450
Star/β-actin
p=0.345
p=0.533
Mdk/β-actin
p=0.111
p=0.493
Meis1/β-actin
p=0.211
p=0.899
DMSO
EG1 50µM
EG1 100µM
0 hours (E12.5)
72 hours

Figure S6

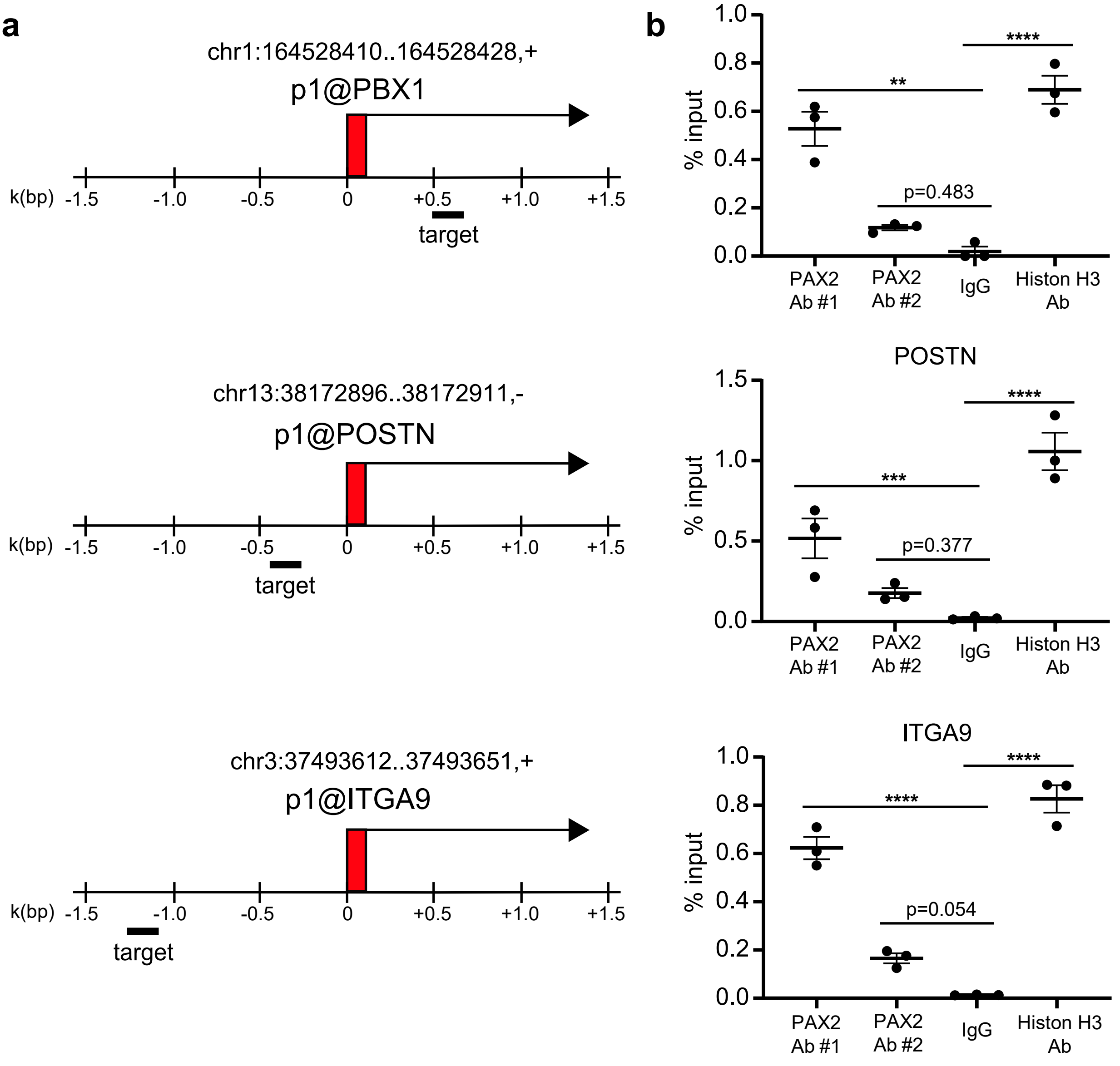
a
chr1:164528410..164528428,+
p1@PBX1
k(bp) -1.5 -1.0 -0.5 0 +0.5 +1.0 +1.5
target
chr13:38172896..38172911,-
p1@POSTN
k(bp) -1.5 -1.0 -0.5 0 +0.5 +1.0 +1.5
target
chr3:37493612..37493651,+
p1@ITGA9
k(bp) -1.5 -1.0 -0.5 0 +0.5 +1.0 +1.5
target
b
PBX1
% input
1.0
0.8
0.6
0.4
0.2
0.0
**
****
p=0.483
PAX2 Ab #1
PAX2 Ab #2
IgG
Histon H3 Ab
POSTN
% input
1.5
1.0
0.5
0.0
***
****
p=0.377
PAX2 Ab #1
PAX2 Ab #2
IgG
Histon H3 Ab
ITGA9
% input
1.0
0.8
0.6
0.4
0.2
0.0
****
****
p=0.054
PAX2 Ab #1
PAX2 Ab #2
IgG
Histon H3 Ab
